# An ethnography of construction and characteristics of curriculum for inheritance of intangible cultural heritage martial arts in universities

**DOI:** 10.3389/fspor.2024.1395128

**Published:** 2024-05-15

**Authors:** Yuanlong Cheng, Nana Guo

**Affiliations:** ^1^ Graduate School/Discipline Construction Division, Cheng Du Sport University, Chengdu, China; ^2^ Department for Improvement of Teachers, China West Normal University, Nanchong, China; ^3^ Department of Sports, Xishan Foreign Language Experimental School, Nanchong, China

**Keywords:** curriculum construction, intangible cultural heritage martial arts, martial arts education, traditional culture, local knowledge

## Abstract

**Background:**

Inheriting excellent traditional Chinese culture is a prerequisite for ensuring the continuity of the cultural genes of the Chinese nation. However, with the historical mission of shaping the national character of the descendants of the Chinese nation, intangible cultural heritage martial arts face the problem of an unclear curriculum content construction mechanism in university inheritance and make it difficult for these martial arts to shoulder the responsibility of cultural inheritance in the era.

**Method:**

Educational ethnography as a research method is conducive to the in-depth exploration of the mechanism of curriculum content construction in the inheritance of intangible cultural heritage martial arts in universities.

**Results:**

Research suggests that the construction of curriculum content for the inheritance of intangible cultural heritage martial arts in universities relies on the form of cooperation between universities and inheritors, and form three models of curriculum content construction: off campus, on campus, and on-campus cooperation. The construction of curriculum content for off-campus inheritors belongs to the “attachment style” model, which is based on the actual needs of the inheritors and the selection of boxing types. The construction of curriculum content for inheritors on campus belongs to the “reshaping” model, which is the inheritor's “simplification and reorganization” based on traditional routines and subjective and objective conditions of inheritance. The construction of course content in school local cooperation belongs to the “integrated” model, which is the reintegration of course content by universities based on their own development characteristics and the characteristics of various martial arts.

**Conclusion:**

In the inheritance of intangible cultural heritage martial arts in universities, emphasis is placed on local knowledge, core skills, cultural traditions, and other characteristics, highlighting the excellence of its cultural inheritance. In the future, the focus of the inheritance of these martial arts in universities should be to cultivate innovative talents who are familiar with both traditional and modern martial arts.

## 1 Introduction

China's excellent traditional culture inherits the continuity of the cultural bloodline of the Chinese nation and bears the responsibility of shaping the national character of the descendants of China. As a typical representative of China's excellent traditional culture, intangible cultural heritage martial arts play an important role in maintaining the national bloodline and shaping the national character, especially in shaping the worldview, values, and outlook on life of contemporary college students, which determine the question of “what kind of person” the young generation of college students should be cultivated into. However, in the process of inheriting intangible cultural heritage martial arts in universities, “martial arts in physical education classes at all levels of ordinary schools tend to enhance students’ physical fitness, cultivate national spirit, and are more inclined toward the ‘utilization’ of martial arts, unable to bear the heavy responsibility of inheriting intangible cultural heritage martial arts” (1), “despite repeated calls from scholars and continuous measures taken by the government and schools, the results have been minimal” (2). Ultimately, the key to the educational inheritance of intangible cultural heritage martial arts in schools lies in the construction of curriculum content, which determines the nature of student cultivation. In addition, the introduction of these martial arts into campuses has attracted the attention of many scholars, and many universities have also offered intangible cultural heritage martial arts courses (3). However, the curriculum construction mechanism in the inheritance of intangible cultural heritage martial arts in universities is still unclear, resulting in the cultural inheritance of these martial arts in universities being in vain. Based on this, the present study examines the inheritance of Sichuan intangible cultural heritage martial arts in universities as a local experience through using participatory observation and in-depth interviews. It studies the process of curriculum construction in the inheritance of intangible cultural heritage martial arts in universities. It then condenses the characteristics of curriculum construction in the inheritance of intangible cultural heritage martial arts in universities, and explores how traditional martial arts culture can be integrated into the modern educational system.

## 2 Literature review

The construction of curriculum plays an important role in school education in China, and a series of policies have been formulated to support curriculum construction. Since 1998, China has continuously adjusted and reformed its curriculum system, gradually establishing a three-level curriculum system consisting of national curriculum, local curriculum, and school-based curriculum. In 1999, the decision was made to deepen education reform and comprehensively promote quality education. In 2001, a new round of curriculum reform was implemented, and in 2010, the National Medium- and Long-Term Education Reform and Development Plan Outline (2010–2020) was formulated. In 2012, the revised national curriculum standards for various disciplines were promulgated and implemented. Although there are over 100 concepts about curriculum, the curriculum began to shift from curriculum development to curriculum understanding after the 1980s. “The essence of curriculum lies in constructing culture” (4). Scholars have proposed a representative curriculum view, where curriculum refers to subject textbooks, teaching plans, teaching objectives, and learning experiences (5). There have also been studies exploring the issue of curriculum views from the relationship between structuralism and constructivism. It is believed that the common goal of both theories is to break through the surface disciplines of knowledge structure and move toward the deep structure of basic concepts in curriculum construction (6). Although these concepts are expressed differently, they all emphasize the issue of what to teach. Researchers have summarized the trends and characteristics of international curriculum reform, including focusing on student development and emphasizing the cultivation of skills needed to adapt to modern society; emphasizing the integration of the curriculum and the mutual integration between disciplines; integrating quality evaluation standards into the curriculum and emphasizing accountability; and the decentralization of educational management power and the centralization of curriculum evaluation power (7). Researchers have also studied the curriculum construction of mixed-age education for young children, believing that the construction of mixed-age courses must adhere to the principles of subjectivity and development, comprehensiveness and diversity, and interest and integration (8). At the same time, some scholars have proposed a constructive view on curriculum culture, which emphasizes that curriculum construction is not a linear program of indoctrination, rote memorization, mechanical stimulation, passive response and reinforcement, closed linear procedures, and rigid evaluation standards and methods, but rather a non-fixed and universal program or method (9). These studies further highlight the diversity in curriculum construction, making the concept of diverse curriculum construction a universal feature. However, with the acceleration of global integration, there have been some changes in the concept of curriculum construction, among which the concept of a multicultural education curriculum is prominent, especially in ethnic minority education. Some scholars advocate the use of “open curriculum” and “multicultural new curriculum models” in curriculum construction, rather than building a new curriculum, but integrating various distinctive cultures into the modern curriculum (10). Researchers have also discussed the theory and strategy of local cultural curriculum construction from an objective perspective. They believe that in local curriculum construction, the interaction between ethnic cultural inheritance and student cultural consciousness should be achieved, reflecting the nature of activity-based and comprehensive curriculum, focusing on theoretical orientations, such as knowledge and skills, processes and methods, emotional attitudes, and values. In terms of strategy, the combination of knowledge logic and life logic can be used to highlight comprehensive cultural events, choose the form of student-centered dialogue, and lead teaching reform (11). Therefore, whether it is the construction of multicultural courses or local courses, it emphasizes the various possibilities of curriculum construction. Curriculum construction is a large concept that includes three steps: “identifying the theme, constructing a knowledge network around the theme; identifying, expressing lasting understanding and writing basic questions; and writing knowledge and skill goals” (12). Although researchers have gained in-depth understanding in curriculum construction, there are still some problems that have been addressed from different perspectives. For example, in response to issues such as detachment and lack of autonomy in curriculum construction, some researchers have conducted specialized research on these issues, further improving the system of embodied curriculum from three aspects: theoretical origins, core features, and embodied curriculum construction. In the construction of embodied curriculum, curriculum construction should be carried out from both horizontal and vertical dimensions (13). Some researchers also believe that contemporary curriculum construction should focus on cultivating students’ expectations of unrestricted thinking and critical thinking, considering the problems of cultural autonomy and autonomy in the old curriculum model (14). Especially in the context of the information age, some scholars have constructed information core literacy courses to solve the problem of not keeping up with the times due to the rapid transformation of the times (15). Some researchers also believe that curriculum construction has shifted from behaviorism to cognitive science in theory (16). From the perspective of cognitive science, these papers reflect on the deep-seated issues of curriculum construction.

In the field of martial arts, Chinese martial arts have always faced many new reforms since modern times, such as the reform from cultivating military war talents to cultivating modern martial arts sports talents, and from traditional ethnic sports to modern sports, so the education is an important aspect of its reform. As a result, many problems of martial arts education have been encountered, and related research is also relatively rich. Under the martial arts education model, martial arts in schools exhibit a certain degree of “educational vitality” (17), which helps “cultivate students’ physical literacy” (18), “shape their national spirit” (19, 20), and “promote aesthetic education” (21, 22). In the new historical context, the simultaneous development of “five educations” (23) has become a new trend in the development of martial arts education in schools. However, martial arts in schools also face various problems as they transition toward the field of education, such as “insufficient motivation after entering school education” (24) and “lack of humanistic spirit” (25); these difficulties in martial arts education in schools mainly manifest in “understanding and practice” (26). As a result, related research is also relatively rich. Some researchers believe that building a unique cultural system for martial arts education in schools, or implementing a curriculum centered on martial arts, is an effective way to achieve the prosperity and development of a martial arts education in schools (27). Some researchers have also introduced the Sport for All theory and suggested that the school martial arts courses should be constructed according to the seasons (28).

However, this curriculum construction is still only at the theoretical level and has not yet entered specific curriculum practice, and the specific teaching results are not very clear. Some researchers have also studied the development of a school-based martial arts curriculum in primary and secondary schools, starting from the social background of “one school, one fist” (29). In order to gain a deeper understanding of the problems in curriculum construction, researchers conducted a survey of 252 ordinary primary and secondary schools in 30 provinces of China, believing that educational content should follow the combination of tradition and innovation, simplicity and systematicity, unity and diversity, fitness and combat, martial arts spirit and patriotism; the content level is divided into fun martial arts, skilled martial arts, and cultural martial arts; the content categories include nationally unified demonstration content, local boxing's characteristics, and female content; the content composition includes bare hands, equipment, theoretical knowledge, among others (30). However, there is no specific plan for Chinese martial arts, so its practicality is insufficient.

At present, integrating ideological and political education into the curriculum has become a new approach for martial arts education in schools, aimed at fostering moral education and cultivating talents. In the context of ideological and political education in the curriculum, it is important to fully explore the ideological and political elements of martial arts and integrate martial arts education with ideological and political education. From the perspective of curriculum ideology, some researchers emphasized that the construction of physical education curriculum ideology is an urgent task for the construction of a strong sports country, the mission of school education, and the growth needs of college students (31). Some studies also suggest that an ideological and political curriculum is in line with school-based martial arts education in terms of educational philosophy, content, and subjects. In the context of this curriculum, the return of the “martial arts spirit” in martial arts education can be re realized (32). There are also researchers advocating for the reform of university martial arts courses, focusing on the essence of these courses, the cultural phenomenon of martial arts, and the cultural consciousness of martial arts courses to carry out ideological and political constructs (33). From the perspective of cultural inheritance, Liu Wenwu believes that the entry of traditional martial arts into the school system is the fundamental strategy for inheriting martial arts culture. Martial arts majors in sports colleges should shift their curriculum content from competitive martial arts to traditional martial arts. Martial arts teachers are encouraged to the people to learn local customs, while the school's teaching authority should prioritize the retraining of martial arts teachers (34). Based on the needs of society and college students, Wu and Lv have developed a curriculum reform plan to response to the problems of single and outdated content, lack of martial arts characteristics in university martial arts courses, as well as the fragmentation, abandonment, dismemberment, and piecing of martial arts curriculum reform. Their proposal has been empirically recognized by students, experts, and evaluations (35). In 1997, Lv and Peng pointed out the main problems faced by the reform of the martial arts curriculum in schools: mainly teachers and textbooks. These issues have been persistently challenging martial arts education. In curriculum construction, the overall goal of “valuing martial arts, promoting morality, and cultivating oneself” should be clearly defined, and the construction of the teaching staff should be strengthened along with the scientific nature of textbook selection (36). From these studies, it can be seen that the construction of a martial arts curriculum exhibits certain historical characteristics and maintains a high degree of consistency with the construction of a national curriculum. Therefore, in the context of cultural inheritance, how to play the role of the inheritance of intangible cultural heritage martial arts has become an urgent issue that needs attention at this time.

In summary, curriculum construction involves discussions and in-depth analyses from different theoretical perspectives and academic disciplines. However, these studies focus on theoretical and objective analyses, ignoring the thematic perspective of curriculum construction. In the field of martial arts, even though some research has examined curriculum reform through surveys, they have not delved deeply into the process of curriculum construction, resulting in curricula not fully meeting teaching needs. In the context of preserving intangible cultural heritage, in particular, the prominent regional characteristics of martial arts show significant differences in the curriculum construction of universities; how to carry out curriculum construction has not been effectively solved. Starting from the perspective of the subject, this study delves into the practice of inheriting intangible cultural heritage martial arts in universities. Through participatory observation and in-depth interviews, it explores the operational mechanism of curriculum construction in the inheritance of these martial arts in universities, summarizes the characteristics of curriculum construction, and further explores how traditional martial arts can be integrated into the modern educational system.

## 3 Materials and methods

This study mainly adopts field investigation methods to collect data through participatory observation and in-depth interviews, including “ethical rules, sampling, questioning, analysis, and memo” (37) to determine “the correct understanding of the research objects” (38). Five universities participated in the observation: Chengdu Sport University; China West Normal University, Neijiang Normal University, Leshan Vocational and Technical College, and Neusoft College in Chengdu. The in-depth interviews followed a semi-structured format in various settings, including offices, training interval, and the accommodation of inheritors of intangible cultural heritage martial arts. The first step of the study was to screen and select the participants. Then, the observation location was determined. Finally, the data were organized and the paper was written completely.

### 3.1 Selection of research object

The inheritance of intangible cultural heritage martial arts in universities is a cultural practice established through cooperation and negotiation between universities and intangible cultural heritage martial arts. As a result, not every university will carry out the inheritance of intangible cultural heritage martial arts. Furthermore, the inheritance of intangible cultural heritage martial arts in universities has characteristics of randomness, so it will be canceled at any time. Based on the instability of the inheritance of intangible cultural heritage martial arts in universities, this study undertook extensive work in the early stages to determine the programs including through official website and snowball methods. Finally, a list of universities and their intangible cultural heritage martial arts projects was completed.

Due to the blurred boundaries of concepts such as Sichuan martial arts, Emei martial arts, Bashu martial arts, there are also some difficulties in determining the object of this study. In light of this, the following measures were taken to clarify the research object and scope. First, the objects of intangible cultural heritage martial arts were clarified. This study only focuses on Sichuan intangible cultural heritage martial arts that have been approved by the state. These include martial arts approved at the national, provincial, municipal, or district level, all of which are within the scope of this study. In the school curriculum, the other martial arts that do not meet the intangible cultural heritage criteria were excluded. This was mainly to avoid non-Sichuan-approved martial arts being included in the study. The reason for this limitation is that universities are a gathering place for multiple martial arts, and the students are from various provinces in China, which is not conducive to the in-depth research of this study. Second, the scope of intangible cultural heritage martial arts was clarified. Because the enrollment of universities is all over the country, so the intangible cultural heritage martial arts projects are mixed from all over the country. Based on preliminary research, it was discovered that almost every university has a martial arts team and foreign intangible cultural heritage martial arts projects, such as Xingyi Boxing, Bagua Palm, Tai Chi Boxing, and so on. Intangible cultural heritage martial arts projects are extremely complex. Moreover, there are over 100 types of Chinese martial arts, and there are hundreds of universities in Sichuan Province. Therefore, the intangible cultural heritage martial arts projects were limited to Sichuan Province to streamline the scope of this study. Third, there are three basic conditions for the inheritance of intangible cultural heritage martial arts in universities: (1) there must be dedicated inheritors or teachers of intangible cultural heritage; (2) it must be an intangible cultural heritage martial arts project approved or recommended by Sichuan Province only; and (3) students must practice with them actively, and not just engage in temporary intangible cultural heritage activities such as cultural festivals and cultural corridors. Only projects meeting the above three criteria are included in the scope of this study.

### 3.2 Selection of interviewees

This cooperation is established through thorough consultation between intangible cultural heritage martial arts entities and universities. Therefore, the inheritance of intangible cultural heritage martial arts in universities has not shown universal characteristics, and only some universities and intangible cultural heritage martial arts entities have collaborated. As a result, this study adopts a snowball approach to search for interviewees effectively. Two types of interviewees were selected as the focus: inheritors of intangible cultural heritage martial arts in Sichuan Province and teachers involved in tangible cultural heritage martial arts in universities. The reason for choosing Sichuan Province is that the enrollment system of universities determines the extremely wide variety of college students, which lead to diverse intangible cultural heritage martial arts. The inclusion of university inheritance teachers is those who have already taken on the mission of inheriting intangible cultural heritage martial arts. Specifically, when selecting interviewees, the interviewees should be those who have carried out intangible cultural heritage martial arts in universities. The teachers interviewed should be in-service university teachers who have implemented or are currently implementing intangible cultural heritage martial arts teaching activities. Specific interview details are shown in Tables 1, 2.

**Table 1:** List of interviews with inheritors of intangible cultural heritage martial arts universities.

Number	Gender	Inheritance project	Teaching methods	Identity of interviewee
CCR—F01	Male	National-level projects	Public physical education class	Representative inheritors
CCR—F02	Male	National-level projects	Leisure physical education class	Representative inheritors
CCR—F03	Male	Provincial-level projects	Public physical education class and martial arts professional class	Representative inheritors
CCR—F04	Male	Provincial-level projects	Public physical education class	Representative inheritors
CCR—F05	Male	Provincial-level projects	Public physical education class and extracurricular training courses	Representative inheritors
CCR—F06	Male	Provincial-level projects	Physical education elective course	Representative inheritors
CCR—F07	Female	City-level projects	Public physical education class	Representative inheritors
CCR—F08	Male	City-level projects	Public physical education class	Representative inheritors
CCR—F09	Male	City-level projects	Public physical education class	Representative inheritors

**Table 2:** List of interviews with teachers of intangible cultural heritage martial arts universities.

Number	Gender	Inheritance project	Identity of interviewee
GXJS—F1	Male	National-, provincial-, and municipal-level projects	University martial arts teacher
GXJS—F2	Male	National-level projects	University martial arts teacher
GXJS—F3	Male	National-level projects	University martial arts teacher
GXJS—F4	Male	National-level projects	University martial arts teacher
GXJS—F5	Male	National-level projects	University martial arts teacher
GXJS—F6	Male	National-level projects	University martial arts teacher
GXJS—F7	Female	National-, provincial-, and municipal-level projects	University martial arts teacher
GXJS—F8	Male	National-, provincial-, and municipal-level projects	Head of University Intangible Cultural Heritage Martial Arts
GXJS—F9	Male	National-, provincial-, and municipal-level projects	Retired university martial arts teacher
GXJS—F10	Male	National- and provincial-level projects	Head of University Intangible Cultural Heritage Martial Arts
GXJS—F11	Male	National- and provincial-level projects	University martial arts teacher
GXJS—F12	Male	National-, provincial-, and municipal-level projects	University martial arts teacher
GXJS—F13	Male	National-, provincial-, and municipal-level projects	University martial arts teacher
GXJS—F14	Female	National-, provincial-, and municipal-level projects	University martial arts teacher

### 3.3 Arrangement of on-site observation points

An important feature of this study is the participation in on-site teaching for the inheritance of intangible cultural heritage martial arts in universities. This is conducted through observation, recording, interviews, drawing, and other methods of on-site teaching. Therefore, determining observation points, introducing these points, and how to carry out systematic observation procedures have become key components of this study.

**Figure 1:**
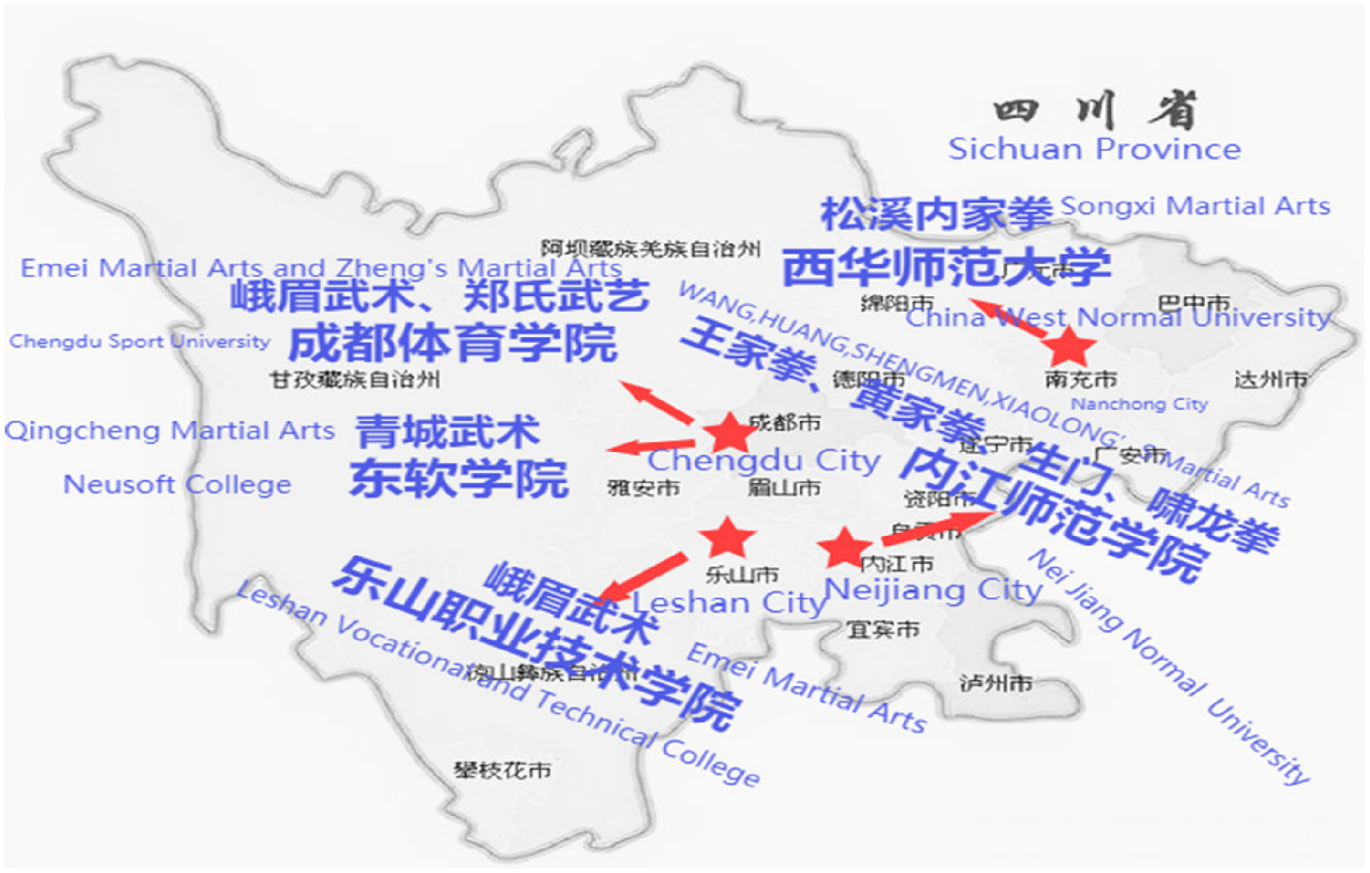
Map of observation points on the heritage site of intangible cultural heritage martial arts universities.

First, the determination of on-site observation points involved several steps. The first step was to determine the types, levels, geographical locations, and representative inheritors of Sichuan Province's intangible cultural heritage martial arts using the China Intangible Cultural Heritage Network (China Intangible Cultural Heritage Digital Museum) and Sichuan Intangible Cultural Heritage Network. The second step was to determine whether the project had been practiced in universities or not by universities' official website. The third step was to identify the level and name of universities in Sichuan Province through the official websites of the Ministry of Education and the government of the People's Republic of China. It should be noted that due to certain misunderstandings among universities regarding concepts such as intangible cultural heritage martial arts and inheritance, our preliminary field research led us to select specific universities conducting intangible cultural heritage martial arts teaching activities as our observation objects. In other words, universities chosen for observation must meet the following conditions. Firstly, there must be the teachers (inheritors) who are implementing in universities. Secondly, there must be a target audience (students) for inheritance of intangible cultural heritage martial arts. Thirdly, there must be specific teaching (or inheritance) activities. All three are indispensable, as shown in Figure 1.

Second, the on-site observation points were summarized. The first on-site observation point was Chengdu Sports University, one of the six sports colleges directly under the former General Administration of Sport of China. It is now a jointly established institution between Sichuan Province and the General Administration of Sport of China and is the oldest sports college in China. The school currently has over 9,300 full-time undergraduate students and over 1,300 doctoral and master's students. The school currently has over 1,200 in-service teaching staff, including over 600 full-time teachers and nearly 280 senior professional titles. Focusing on undergraduate education, the university has 21 teaching and research units offering 22 undergraduate majors spanning 7 university disciplines: education, medicine, literature, management, economics, art, and history. The school has first-level disciplines authorized for doctoral degrees in sports, as well as for master's degrees in sports, clinical medicine, integrated Chinese and Western medicine, and journalism and communication. There are also 14 second-level disciplines authorized for master's degrees, as well as four master's degree authorization points in sports, traditional Chinese medicine, journalism and communication, and art. The school established the Department of Martial Arts and Heavy Sports in 1959, the Department of Chinese Martial Arts in 1992, and the Martial Arts Academy in 2017. The martial arts college of the school has received many awards, including the National Youth Martial Arts Routine Training Base and the Sichuan Intangible Cultural Heritage Research Base. The college's martial arts and traditional sports majors have been approved as characteristic majors, key disciplines, and national characteristic majors. The school established the Chinese Academy of Arts in May 2020 and was jointly designated as one of the first batch of provincial key Chinese cultural research institutes by the Propaganda Department of the Sichuan Provincial Party Committee, the Sichuan Provincial Department of Education, and the Sichuan Provincial Federation of Social Sciences. It aims to uphold and implement traditional Chinese culture in the country and Sichuan Province, focusing on building and promoting the Chinese spirit, reflecting the characteristics of Bashu, and jointly shouldering the historical mission of promoting excellent traditional Chinese culture with the school's martial arts academy. Emei martial arts and Zheng's martial arts are key projects in the curriculum construction of the school. Currently, two intangible cultural heritage martial arts projects have already been included in the undergraduate and graduate training systems.

The second on-site observation point was China West Normal University, a key provincial university in Sichuan Province, founded in 1946 and located in Huafeng Town, Shunqing District, Nanchong City. The school currently has 82 undergraduate majors, 19 first-level disciplines authorized for master's degrees, 13 categories authorized for master's degree programs, 6 disciplines jointly training doctoral students, and 1 postdoctoral innovation practice base. The school currently has more than 35,000 undergraduate, graduate, and international students. The school mainly focuses on teacher education, with over 2,700 faculty and staff, including over 1,000 teachers with senior professional titles and over 1,900 teachers with doctoral and master's degrees. The school has two campuses, one under construction, covering a total area of 4,000 acres. When Songxi Boxing entered the school in 2010, it went through two stages: inviting in and stabilizing. In 2010, Professor Zheng Zhigang from the School of Physical Education at West China Normal University invited Li Hanguang, a folk inheritor of Songxi Fist, to carry out extracurricular inheritance work at the school on weekends. The inheritance method of “please come in” continued until 2015. After 2015, Professor Wu Panwen from the School of Physical Education of West China Normal University carried out the inheritance work of Songxi Boxing in universities. Wu Panwen graduated from the Department of Biology at Qufu Normal University in 2002 and the School of Life Sciences at West China Normal University in 2006. After obtaining his master's degree, he worked in the Graduate Department of West China Normal University. During his studies in Nanchong, Wu Panwen studied under Huang Yanzhong, the 12th generation inheritor of the folk Songxi Boxing. He began to systematically learn Songxi Boxing and graduated with a PhD in Ethnic Traditional Sports from the School of Martial Arts, Shanghai Institute of Sports in 2015. After graduating with a PhD from the Shanghai Institute of Physical Education, he transferred from the Graduate Work Department of West China Normal University to the School of Physical Education of West China Normal University to work in sports and martial arts teaching. He is a master's supervisor and also responsible for teaching martial arts in public physical education classes for ordinary college students. In addition, the school has established an intangible cultural heritage martial arts training base to carry out cultural inheritance and scientific research on Songxi Boxing for all teachers and students.

The third on-site observation point was Neijiang Normal University, which began offering higher education in 1956 and was upgraded to a university in 2000. The university covers an area of 3,115 acres and is a full-time undergraduate institution under the supervision of the Sichuan Provincial Government. The school is located in an important node city on the main axis of the Chengdu Chongqing Economic Circle. The school integrates graduate education (joint training), regular undergraduate and vocational education, continuing education, and international student education. It has 19 secondary colleges and 64 undergraduate majors, and currently has more than 17,000 full-time undergraduate and vocational students, international students, and graduate students. The school currently has 1,498 faculty members, 1,165 full-time teachers, 454 senior and associate professional titles, and 1,067 master's and doctoral students. The school was approved to establish the Sichuan Emei Martial Arts Culture Popularization Base in 2014, mainly focusing on the research of Emei martial arts culture education as well as the excavation, protection, inheritance, and promotion of Emei martial arts culture.

The fourth on-site observation point was Leshan Vocational and Technical College, located between Leshan Giant Buddha Scenic Area and Mount Emei. It is a double holy land of world cultural heritage and natural heritage, adjacent to the Qingyi River in Suji New Area. The school was established in 2002 by the Sichuan Provincial People's Government and Leshan as a comprehensive full-time public higher vocational college. It has a 70-year history of education and offers disciplines such as medicine and health, finance and tourism, machinery and electronics, and new energy. The school covers an area of over 800 acres, with over 600 faculty members and more than 14,000 full-time students. The school has a strong martial arts atmosphere, mainly through public physical education classes, specialized elective courses, and international students (Laos, Vietnam, and other countries) to carry out Emei martial arts. The teaching content mainly focuses on Emei Fist, and there are approximately 1,000 students participating in teaching activity.

The final on-site observation point was Chengdu Neusoft University, a private ordinary higher education institution approved by the Ministry of Education and funded by Neusoft. In 2021, it became the first university in Sichuan Province to pass the undergraduate teaching evaluation of ordinary higher education institutions. The school is located near Qingcheng Mountain 5A Scenic Spot in Dujiangyan Irrigation Project, covering an area of 528,500 m^2^. The school has 5 disciplines, namely engineering, literature, management, art, and medicine, 31 undergraduate majors, and more than 20,000 students. The national intangible cultural heritage martial arts project, Qingcheng Martial Arts, is an elective course at the university. There are one or two classes per semester, with approximately 50 students in each class. The teaching content mainly focuses on Qingcheng Martial Arts Golden Knife Shedding and Qingcheng Tai Chi Health Preservation.

Thirdly, about the content and records of on-site observations. Given that on-site observations mainly involve individuals, events, and objects, the main focus of observation is on aspects such as content and methods of inheritance, venue facilities, student attitudes, and teacher-student interaction during teaching sessions. Observation not only includes the functional responsibilities, appearance, and language expression of inheriting and inheriting teachers, but also involves the teaching procedures of intangible cultural heritage martial arts, student physical practice, and interactive exchanges. At the same time, attention is given to public spaces, venue facilities, and equipment related to intangible cultural heritage martial arts teaching. Regarding recording methods, we ensured the completeness of teaching records to the greatest extent possible and adopted multiple parallel methods for recording. First, various shorthand methods were used to record the teaching site, and the entire process of teaching is recorded in the form of field diaries. Second, sound data from the teaching sessions were recorded through using recording equipment, and these recordings are promptly converted into text. Finally, the entire teaching process was filmed through using camera equipment and then timely transcribe the teaching process into text.

**Figure 2:**
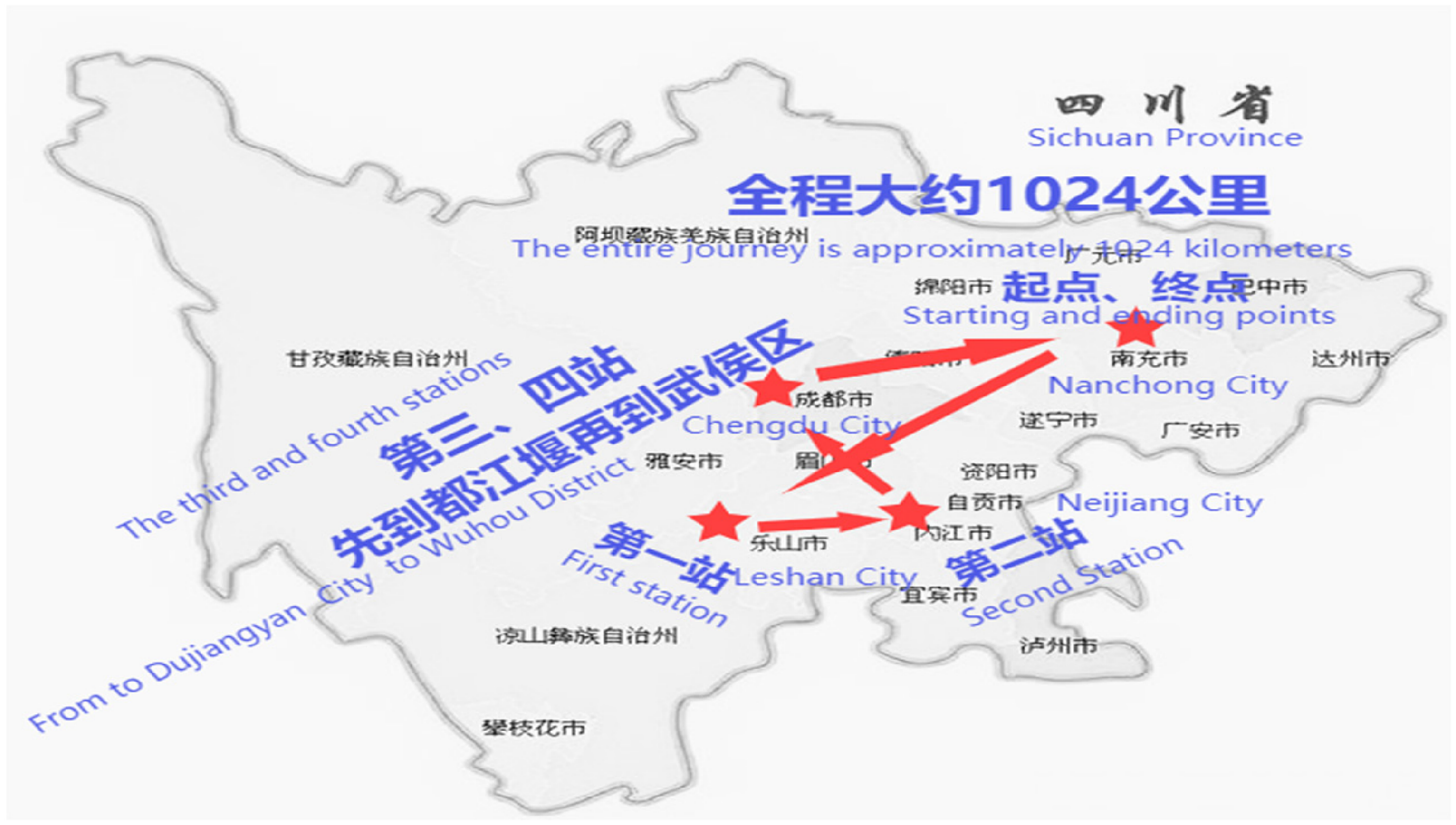
Tour of observations on the inheritance of intangible cultural heritage martial arts universities.

Fourth, the on-site observations were scheduled. The inheritance of intangible cultural heritage martial arts in universities is mainly based on regional martial arts, with a concentration of universities in Chengdu, Neijiang, Leshan, Nanchong, and other areas. They are relatively concentrated in the central area of Sichuan Province, with a distance of approximately 300 km between each university, and a total distance of approximately 1,024 km. This distance increased the difficulty for participation observations to some extent. As a result, the study started from Nanchong city, where the researcher was located. And the researcher used a car as means of transportation to travel back and forth between Nanchong, Leshan, Neijiang, and Chengdu, as shown in Figure 2.

### 3.4 Analysis and organization of research materials

This study involves various forms of data, including interview materials, on-site observation diaries, video and recordings, photographs, and activity materials. Therefore, significant time is required for data processing. Given the nature of these materials, the first step is to write an education log every day, documenting the materials seen and heard that day, and making notes and reflections on the materials during the writing process. Second, on-site recording materials, video materials, interview materials, and temporary conversations are transcribed to form written texts. Once again, it is necessary to review and code the text materials and create a memo during the coding process. Thematic coding is then used to classify the text materials and develop thematic analysis units. Finally, the paper is completed based on the thematic units. In the process of collecting, categorizing, analyzing, and writing, the memorandums of writing is always regarded as an important way to promote the next step of research. Through continuous reflection, the problems are formed and deepened. Ultimately, an educational ethnography was completed.

### 3.5 The identity of the researcher

In scientific research, the identity of researchers should be ignored as much as possible to enhance objectivity and reproducibility. However, in anthropological research, active participation in observation is important, which will inevitably impact the research scene and the researchers. Therefore, achieving the objectivity of observational data while ignoring the influence of researchers poses a challenge in field research. It is necessary to ignore the observer effect of researchers and achieve a true understanding of the research object. This study adopts the several measures to maintain a relationship with the subjects. First, before formal field investigations, researchers establish contact with the inheritors or university faculty who preserve inheritance. An interview is conducted first, followed by long-term communication to prevent discomfort to the inheritors or inheritors caused by unauthorized visits. This also narrows the distance between researchers and subjects, eliminating their guard mentality and encouraging a healthy relationship. For most universities, as martial arts belong to a common profession, there are many similar research topics, and there are no obstacles encountered when entering on-site teaching. Second, for universities that are familiar with inheritance teachers, they can enter through full participation in student training according to their training time. Third, due to the alumni relationship between the researchers and the inheritors of many universities in Sichuan Province, the identity of the researcher are referred to as “brothers” or “uncles” and “students”.

### 3.6 Trustworthiness for research

To ensure the richness and credibility of our data, we engaged in long-term participation with students between 2005 and 2024 during the research phase. In the writing stage, we used member checks to increase the credibility of the study. We provided participants with summaries of our analyses upon completion of the manuscript for feedback. To guarantee external validity, we used thick descriptions. Our research not only focused on positive examples, but also on negative ones to gain a deeper understanding of the problem. We used triangulation methods such as focus groups and documentary analysis, and described the project context, its characteristics, and the selection of participants.

### 3.7 Ethical considerations in research

In anthropological fieldwork, ethical considerations are also an important aspect, as the fieldwork involves deep-seated issues of human nature. As the research progresses, it is no longer a process of data collection, but involves issues such as people's emotions, attitudes, and values. Therefore, protecting the privacy of these individuals is crucial. This study adopts several measures to avoid ethical issues. First, before entering the field, active communication with the participants is undertaken to obtain their consent. Second, it is not necessary to make excessive value evaluations of the individuals’ situation, but to interact and communicate with them as listeners. Once again, it is important to maintain an impartial attitude with the participant and not examine their behavior and cultural phenomena. Finally, in the writing stage, sensitive information related to the research participants will be processed in accordance with academic standards. Personal protection will be provided for the participants, and the role of researchers has been minimized.

## 4 Findings

Education plays an important role in shaping generations. “Education is a key means of enabling individuals to unleash their enormous inner potential, achieve supernatural transformation, establish effective connections between enormous needs and broad openness, and shape themselves correctly” (39). The curriculum content implemented in school education is the key to shaping an individual's future. Intangible cultural heritage martial arts is a body culture accumulated by the Chinese culture for thousands of years, so the martial arts contain profound survival wisdom and life experience. From the perspective of education’s in shaping individuals, the inheritance of intangible cultural heritage martial arts in universities passes on the genes of China's excellent traditional culture. The people cultivated are those given the wisdom and genes of the Chinese culture, rather than those with other cultural attributes. Therefore, constructing the curriculum is the key to the inheritance of intangible cultural heritage martial arts in universities. In the case of Sichuan’s intangible cultural heritage martial arts, the focus is on simple and practical course construction, emphasizing basic skills and martial arts routines. Based on the specific situation of the inheritance of intangible cultural heritage martial arts in Sichuan universities, three types of curriculum construction have been formed: curriculum construction based on internal inheritors; curriculum construction based on external inheritors; and curriculum construction based on inheritance base construction.

### 4.1 Construction of curriculum content based on inheritors on campus

There are two types of inheritors on campus: those who have a teacher-student relationship with intangible cultural heritage martial arts and those who are ordinary martial arts teachers. For inheritors with a teacher-student relationship, more emphasis is placed on simplifying the original content to make the inherited content more suitable for the physiological characteristics of students. On the other hand, ordinary martial arts teachers mostly choose representative intangible cultural heritage projects as inheritance content. For example, take the inheritor of Songxi Boxing, who has a teacher-student relationship. When the inheritors taught the Songxi Boxing in universities, they did not completely copy the content and teaching mode of the folk. Instead, they reorganized and rearranged the content according to the specific situation of the university. They simplified the contents such as boxing, sword, and tiger tail whip, and they changed the originally complex content into a clear hierarchical routine system. From the several martial arts routines currently taught, it can be seen that Songxi Boxing has significantly simplified the content. Why does Songxi Boxing require a simplified routine? CCR-F01, an inheritor of Songxi Boxing at a university, explained it this way: “In the past, I had a lot of practice content, but it was very tiring, and now I don't teach it this way. Some types of boxing are not recorded in that book (“ Songxi Neijia Boxing”) (31 October 2021, Field Diary, CCR-F01).

The simplification and creation of routines is not simply a random combination of routines, but a reintegration and innovation based on the original martial arts, aimed at preserving the core techniques of Songxi Fist. In other words, although Songxi Boxing selects course content through simplified routines in the inheritance of higher education, these routines are the core technologies of the boxing genre. In this sense, the simplified routine does not deviate from the tradition of Songxi Fist, but it undergoes a series of simplification and experimentation processes. For the simplified process of Songxi Fist, the CCR-F01 said, “Songxi Fist Enlightenment Fist was created with great effort by Master Huang and me” (24 October 2021, Field Diary, CCR-F01) and “Songxi Guiyi Fist was created by Master Huang and me. When I was practicing, Master Huang decided which action to use, and thus became the current Guiyi Fist routine” (19 June 2022, Field Diary, CCR-F01).

These created Songxi Fist routines are not created arbitrarily without proper organization, but on the basis of ensuring a certain connection between fists, which facilitates the transfer of movements between different types of fists and preserves certain similarities in movements between routines. At the end of a training session, the inheritance teacher revealed the connection between routines: “Enlightenment Fist and Guiyi Fist are a progressive process, and many movements are reflected in the two types of punches. The Songxi Fist routine we are practicing now is based on the previous one, which used to have a lot of repetitive content. Now, we have deleted these routines. The reason why the previous routines were complex was because they were taught to students in the past. Waiting for students to practice is a tuition fee, and if it's too simple, it won't attract students, so the routine is made up very long. The current routine is not specifically designed for charging” (31 October 2021, Field Diary, CCR-F01).

Creating routines is challenging, as each action needs to be carefully selected to better reflect the technical style of Songxi Fist. Despite this difficulty, it is important that the created routines are understandable not only to the veteran boxer himself but also to others. “The Harrier Fist is the most comprehensive routine, almost including all the movements of the Songxi Fist, and the requirements for footwork are not strict. The Harrier Fist can only be understood by experienced boxers, and was given a low score by the referee when participating in competitions” (19 June 2022, Field Diary, CCR-F01). Based on ensuring the created routine can be understood, the primary goal is to facilitate the teaching of college students, helping them to get started. The inheritance teacher said, “The Songxi Enlightenment Fist had an introductory routine before, and during my teaching, I felt that this routine was not like an introductory routine” (3 July 2022, Field Diary, CCR-F01).

Before the creation of the Songxi Enlightenment Fist, the Songxi Six Step Fist was used as an introductory routine. This routine has long and difficult movements, making it difficult for students to master in inheritance. After the creation of the Songxi Enlightenment Fist, it became an introductory routine for Songxi Fist. The movements are relatively simple and more suitable for new students to learn. In terms of the difficulty of the above two routines, students need to complete one semester to master Songxi Six Step Fist, while the current Songxi Enlightenment Fist can be mastered in half a semester. The decision to simplify these routines came from the “awkwardness” experienced by practitioners in the early stages of training. Because the inheritors of intangible cultural heritage have practiced competitive martial arts before practicing Songxi Boxing, they clearly feel uncomfortable when transitioning to Songxi Boxing. The stark contrast between traditional and competitive martial arts highlighted the need to resolve the “awkward” issue of Songxi Fist. After a training session, the inheritor teacher also had a discussion with the author specifically about the “awkward” problem of Songxi Fist. The inheritor teacher also said, “I have practiced competitive martial arts and some other traditional routines before, but I always feel awkward when practicing Songxi Fist” (30 October 2021, Field Diary, CCR-F01).

To test the effectiveness of the newly created boxing techniques, the inheritors of intangible cultural heritage martial arts conducted special competitions experiments using the simplified boxing routines. These achieved satisfactory results in the competitions. The inheritor said, “The Guiyi Quan that the advanced class is practicing now is a very good boxing technique. I have tried it twice in competitions and achieved good results each time. Now, I don't want Songxi Quan to participate in competitions, so it requires difficult movements, with each difficulty given a point. Many traditional boxing techniques are now taking the path of competitive techniques, but Songxi Quan does not. It has always adhered to traditional things. If students make some competitive movements during practice, I will be corrected Come here” (On 30 October 2022, Field Diary, CCR-F01). The competition experiment mentioned by the inheritance teacher was for martial arts routines for college students in Sichuan Province in 2018 and 2019. Students achieved good rankings in the competition, and the simplified teaching effect was well verified.

There is a fundamental difference between the routine creation and folk creation of Songxi Boxing. There is a mutual connection between different types of boxing, which not only reflects the style characteristics of boxing, but also preserves the core techniques of boxing. From beginning to end, there is a strong coherence, and the techniques are more like a series of attack and defense movements in a technical attack. For example, how to attack the first action, when the enemy escapes, followed by the pursuit of the second action, and so on; the technical attacks are intertwined. In terms of preserving the technical style, each technique originates from the ancient techniques of Songxi Fist, or techniques using the Songxi Fist style are selected for creation. In terms of style, Songxi Boxing effectively protects the style of boxing in the inheritance of higher education, which is a further refinement of core techniques. As explained by the inheritor of intangible cultural heritage martial arts, “Songxi Guiyi Fist is quite classic, and I have also shown it in other places. Others wanted me to teach it, but I did not. He said that only Xihua Normal University has the Songxi Guiyi Fist, and there are no other places” (26 February 2022, Field Diary, CCR-F01).

Maintaining the original flavor of traditional martial arts is a major challenge in its development and has always been a focus of debate in various fields. Some people even have doubts about how intangible cultural heritage martial arts can maintain their original flavor. As for the creation of Songxi Boxing in universities, they are more focused on refining the core techniques of their martial arts, and the style of their martial arts has not changed. In this sense, the core technology is the original flavor of the boxing, which breaks through the narrow understanding that the boxing must remain the same as the original boxing. Qiu Pixiang once mentioned the issue of core techniques in traditional martial arts in an interview, emphasizing the need to leverage the “corner” of traditional martial arts, which emphasizes the core techniques and techniques of traditional martial arts (40). Songxi Boxing embodies the original inheritance concept by retaining the style of its core techniques. It is worth noting that although inheritors in universities attach great importance to the preservation of the core skills of Songxi Boxing, intangible cultural heritage martial arts inevitably change with the habits of different practitioners. Students will make certain adjustments during practice based on their habits, bodily sensations, and other factors. Therefore, it is necessary to approach the issue of the authenticity of boxing with rationality. Undoubtedly, retaining core technologies is an effective way. This is also where the inheritance teacher is very satisfied and proudly says, “Only China West Normal University can have the return to one fist.” Therefore, the classicism of Songxi Fist is not entirely authentic, but it can still achieve maximum modern innovation, and the created routines have also been recognized by others, which means achieving the integration of tradition and modernity. Innovation itself is the process of constantly refining essence, and the classic style of boxing should also be considered. Creation itself is art: “Songxi Guiyi Boxing is an adaptation of the existing 11 sets of movements of Songxi Boxing. We are thinking about those movements every day. Songxi Boxing not only has offensive and defensive skills, but also has some solutions.” “By practicing Songxi Fist, one can better evaluate other punches. In comparison, after practicing Songxi Fist, one does not want to watch other punches” (5 March 2022, Field Diary, CCR-F01).

This highlights that the martial arts routines of Songxi Boxing in university inheritance are not the result of random patchwork, but rather a combination of all the classic movements of Songxi Boxing. It also shows that the attack, defense, and solution of Songxi Fist have been preserved. In this sense, “professional things should be done by professional people” is a very appropriate description of the creation process of Songxi Fist. The practice of randomly creating routines online has been criticized by inheritors. The inheritor said, “I have a friend in Wudang Mountain, where there are also many practitioners of Songxi Fist, but they are all just superficial and learned online. We don’t know the connotation of Songxi Fist, but we are different here. We explain every action very clearly” (5 March 2022, Field Diary, CCR-F01). To ensure the classic nature of the martial arts and the authenticity of the inheritance content, the number of inheritors who will pass it on are the key considerations of inheritor teachers. However, the inheritance teacher said, “Only a small number of people are passing the Guiyi Fist, and they don't want to pass it on a large scale. The number of people should not exceed 10, and there will be various conflicts when there are more people. That is not to say Songxi Fist is conservative, but I'm afraid that some people in the folk will randomly arrange for economic benefits after learning it. Some people will lose out in competitions because there is no difficulty, so many people have changed their movements in order to participate in competitions. We only inherit these things in universities, and outsiders will definitely not learn them” (21 May 2022, Field Diary, CCR-F01).

For inheritors of intangible cultural heritage martial arts in universities, the inheritance of Songxi Boxing does not depend on numbers, but on having trustworthy students. This is also a difference between university inheritance and folk inheritance. In the folk tradition, what may be contested is of economic value. If it is advantageous, it can be taught, even if it destroys the original appearance of the martial arts. However, in universities, inheritors will preserve the original appearance of the martial arts in a new form and pass it on to students. In universities, the inheritance of martial arts routines by inheritance teachers is determined by the learning capacity of college students. Students cannot learn dozens of martial arts routines within a limited period, nor do they have the energy and time to do so. The creation of this routine can be seen as innovation from inheritance teachers, integrating dozens of tedious routines into one routine, ensuring its smooth inheritance in colleges and universities. Although the routine is not the original one, it retains the essence of Songxi Boxing.

### 4.2 Curriculum content construction based on extracurricular inheritors

From an educational anthropology perspective, local textbooks are important curriculum resources for educational implementation, and can be transmitted via principles such as “cognition, emotion, social identity, and individual cultural adaptation” (41). Intangible cultural heritage martial arts exist more as local knowledge and belong to a local cultural tradition, thus possessing the characteristics of local curriculum resources. However, they are heavily based on the characteristics of the boxing styles and personal cultivation of the inheritors. Compared to the course content chosen by university teachers on campus, the construction of content based on external inheritors is more distinct. The construction of course content is based on hot topics such as martial arts, students’ abilities, and course continuity. For example, when inheriting Qingcheng martial arts in universities, the focus is on its local cultural characteristics. CCR-F03, the national inheritor of Qingcheng Martial Arts, introduced the entire process of constructing the content of Qingcheng Martial Arts curriculum: “We hope that college students can feel the traditional culture. When they arrive at Qingcheng Mountain, they can practice Qingcheng Wushu, and feel the local culture. For college students, cultural confidence and cultural identity are still helpful in various aspects. Their school is dedicated to sports such as running jump shots every day, or athletics or track events, which may not be very rich or relatively simple, while martial arts are relatively supplementary” (12 January 2023, Field Diary, CCR-F03).

Human ecologists believe that “humans are influenced by the environment, and at the same time, they also influence the environment, which is achieved through interdependent systems formed between humans, the natural environment, and members of society” (42). The ecological environment requires more human creation, because students have a short time in school. Even if they are in a specific cultural environment, it is difficult for them to perceive regional culture. However, by creating specific cultural scenes and offering reasonable guidance, students can better understand regional culture. The introduction of Qingcheng martial arts into universities is to guide local culture, create a cultural inheritance environment, and encourage students to better understand intangible cultural heritage martial arts, enabling them to promote culture. So why do inheritors of intangible cultural heritage martial arts emphasize the importance of regional culture? After further research, we know that the universities do not lack sports projects or martial arts related teaching content, but rather lack curriculum content with local characteristics. The regional cultural characteristics are the competitiveness and advantages of Qingcheng martial arts. “For ordinary martial arts, there are more students. But our martial arts are a supplement to local culture, or a supplement to martial arts culture. After all, you are in Qingcheng Mountain in Chengdu. It may also represent a local culture, not just competitive martial arts. Competitive martial arts, as mentioned earlier, are very good. Its main focus is on competition, and we emphasize cultural development” (12 January 2023, Field Diary, CCR-F03).

As a form of local knowledge, intangible cultural heritage martial arts reflect more of the local culture, which is also an important value for its entry into university inheritance. It can fill gaps in mainstream educational knowledge, and this is also a cultural advantage. From an anthropological perspective, education is a broad concept that not only includes school education, but also informal learning, such as family education and social education. It is not only a comprehensive forms of education, but also a broad educational attitudes. From this perspective, family or social education does not impart knowledge to students through fixed class hours, but through customs, physical experience, and even myths, legends, and stories. Intangible cultural heritage martial arts have educational value because they contain survival experience and wisdom with regional customs and traditions, and are cultural traditions accumulated within a local scope. Therefore, the inheritors of intangible cultural heritage martial arts are very proud to say “as a supplement to local culture.”

When the inheritors of intangible cultural heritage martial arts understand the importance of local culture, the dissemination of local knowledge becomes the main focus of content construction. Specific inheritance content will then be selected based on the school’s circumstances. In the construction of the inheritance content of intangible cultural heritage martial arts in universities, it is not about blindly choosing one or two routines as course content, but about making content choices based on the school’s specific educational environment. This mainly depends on the students' interests, learning hours, and their training goals. The inheritor of Qingcheng Martial Arts described the main factors to consider when choosing inheritance content and gave a vivid example of course selection: “It is unrealistic for students to learn a lot of things for 32 h in a semester. I think Tai Chi is very practical. My students at the School of Business are in the leisure and sports field, and most of them come out in professions related to sports, tourism, and leisure sports. The graduate needs leadership, team building, and the health group around him. There is a greater demand in society for Tai Chi, so they can use Tai Chi activities during team building. The classes here were quite tight, I used a short fist and a set of short Tai Chi to let them all feel it. Because they are not professional learners after all, so I combine this fact, martial arts and Tai Chi are half and half. I let them fully experience the characteristics of Qingcheng Mountain martial arts, he can strengthen his physical fitness, and after working in society, he can have some applications” (12 January 2023, Field Diary, CCR-F03).

The inheritors of intangible cultural heritage martial arts are extremely cautious in constructing inheritance content in universities, which is also an extremely complex cultural decision. They must consider various aspects, including the nature of the curriculum, student interests, practicality, students’ martial arts foundations, and employment requirements before finalizing the inheritance content. The construction of content for the inheritance of intangible cultural heritage martial arts in universities is a targeted cultural selection process, aimed at providing tangible benefits to students. Given the limited time students have to practice in universities, usually just one semester, it is not realistic to arrange excessive teaching content.

*“‘Simplicity and practicality’ have become the basic principles followed in the inheritance of intangible cultural heritage martial arts in universities. When constructing the inheritance content of intangible cultural heritage martial arts, it also focuses on some hot topics, such as explaining topics that students are interested in, answering social hot topics that students are concerned about to alleviate students’ misunderstandings of martial arts, and correct their biases towards Chinese martial arts. I will not only teach a set of punches. Actually, the culture is very important behind the skills. I will break down each action and explain to him how the technique is used. Of course, I will always emphasize that it's not about letting you know and then fighting or trying. I hope you understand this culture. The difference between martial arts and dance, as well as acrobatics, lies in the meaning of offensive and defensive techniques. Otherwise, what is the difference”(12 January 2023, Field Diary, CCR-F03).*

Intangible cultural heritage martial arts are also traditional martial arts in essence, and the martial arts are the basic premise of its existence. However, the inheritors of intangible cultural heritage martial arts do not only consider its martial arts. They also explain the cultural significance behind the intangible cultural heritage martial arts actions to students, so that they can understand it from a broader perspective. From a cultural perspective, it is important to help students understand that intangible cultural heritage martial arts not only focus on fighting, but also reflect traditional Chinese culture. From a cultural cognition perspective, students mainly rely on fragments of new media networks to learn the intangible cultural heritage martial arts; thus they do not have a deep understanding of it, and they often cannot discern malicious events on the Internet. Therefore, as inheritors of intangible cultural heritage martial arts, they must clarify online misconceptions to students from a more professional perspective, which can resolves various misunderstandings about intangible cultural heritage martial arts. Explaining offensive and defensive techniques realistically without exaggeration can establish an understanding of the principles of martial arts for students.

Technical combat is an essential part of Chinese martial arts; however, after the use of cold weapons, the functions of Chinese martial arts have undergone significant changes. The question of whether Chinese martial arts can fight has become a social issue. In addition, with the hype of various online content, the stigmatization of Chinese martial arts “flower boxing and embroidered legs” has been infinitely amplified, so the stigmatization added the negative portrayal of Chinese martial arts. In this context, as disseminators of intangible cultural heritage martial arts in universities, it is natural for inheritors to teach the correct concept of martial arts to students, so that they can calmly view the issue of “no fighting” in Chinese martial arts. This also fully demonstrates that the inheritor of intangible cultural heritage martial arts is not only a disseminator of Chinese culture, but also a legitimate shaper of Chinese cultural concepts. Therefore, when constructing the content, they firmly choose to be a correct defender of Chinese martial arts culture.

This shows that this is a result of the interweaving of diverse values, which also indicates that the inheritance concept of intangible cultural heritage martial arts in universities has undergone profound changes. The inheritance of intangible cultural heritage martial arts in universities is not only a superficial technological development, but also an awakening of cultural functions. In addition, inheritors of intangible cultural heritage martial arts have also seen that they should not only focus on advanced academic theories, but also they should engage with reality and keep up to date to ensure cultural vitality.

### 4.3 Course content construction based on base construction

In addition to using both on-campus and off-campus inheritors to construct content, there is also a “school local cooperation” curriculum model for the inheritance of Sichuan intangible cultural heritage martial arts universities. This model requires the integration of local intangible cultural heritage martial arts resources to determine its specific curriculum content. Particularly in universities with official national approval and licensing, curriculum content is often determined by integrating on-campus and off-campus projects at all levels (including national, provincial, municipal, district, and county levels). Some universities may even condense one to two sets of school-based curriculum resources in dozens of intangible cultural heritage martial arts projects, forming a complementary curriculum of “off-campus resources” and “school-based resources.” Compared with the curriculum construction methods above, this model plays a decisive role on base construction, influencing content selection, determination, implementation, and evaluation. However, the inheritors of intangible cultural heritage need to construct the curriculum content in a complex power structure. Universities with a focus on “base construction” first need to meet the needs of their own inheritance bases, complete various university cultural inheritance base tasks, and meet the review requirements from relevant approval departments. Therefore, curriculum construction has multiple values and is also relatively complex. As a result, curriculum construction based on the inheritance model of intangible cultural heritage martial arts university inheritance bases has higher requirements. When selecting and determining course content, not only are collective efforts needed, but various inheritance resources need to be considered both on and off campus.

Specifically, the curriculum content inherited by Sichuan's intangible cultural heritage martial arts universities focuses mainly on local martial arts, especially on its inherent cultural value. For example, Neijiang Normal University hires inheritors as visiting professors who are inheritors from the national, provincial, and municipal levels. However, due to the time constraint, there requirements for the construction of curriculum content in intangible cultural heritage martial arts are limited. The time constraint prevent the universities to simultaneously teach all of the martial arts activities beyond intangible cultural heritage. Therefore, only the historical origins of intangible cultural heritage martial arts are taught. “I was sitting down to talk about theory. The first class talked about the history of our Peng Family Boxing, how it came about and how it was passed down” (On 9 January 2023, Field Diary, CCR-F07).

Another inheritor of intangible cultural heritage martial arts at the city level has also been invited by the school to be a visiting professor and teach the theoretical content related to martial arts: “The school calls us to give lectures, and each sect teaches according to their own sect. The martial arts of our sect are different from each other. Our sect and other sects are giving lectures, which are divided into two parts. In the classroom, there are cultural lectures, and each sect teaches different things, such as origin, characteristics, and inheritance” (On 10 January 2023, Field Diary, CCR-F08). This shows that the curriculum content construction mainly focuses on explaining the school’s origin, inheritance, and characteristics; however, this construction is also the result of a joint discussion between the school and the inheritors. From the universities’ perspective, the inheritance of intangible cultural heritage martial arts should mainly emphasize cultural inheritance, rather than imparting intangible cultural heritage techniques. Inheritors of intangible cultural heritage martial arts choose these contents because curriculum construction is determined by the school's cultural regulations and teaching methods. Multimedia classrooms naturally focus on theoretical teaching, and the history of boxing is the best choice. Another reason is that the inheritors of intangible cultural heritage martial arts are older and have difficulty demonstrating movements, making theoretical teaching a better way to leverage the cultural advantages of intangible cultural heritage martial arts. However, in curriculum construction, the emphasis is on highlighting the characteristics of the martial arts genre. For example, in the teaching of Wude, the inheritors have strong characteristics of the martial arts genre through the form of four songs:

“*I started with four songs in class. The first is the song of apprenticeship, the second is the song of practice, the third is the song of thugs, and the fourth is the song of martial arts. Actually, we only attended one class at the teacher's college. We only took one class for inheritance, and we only needed one class to pass on our sect. Each sect has actually gone there only twice, and we have only gone there twice. Because you have too many traditional martial arts, you need to influence other formal courses. If you want to learn from various schools of martial arts, such as Xingyi Quan, Pengjia Quan, Wangjia Quan, Huangjia Quan, and Xiaolong Quan), it will inevitably affect its teaching, and you don't have time to arrange it. Therefore, it is impossible for it to go in large numbers” (On 10 January 2023, Field Diary, CCR-F08).*

However, due to the limited number of class hours allocated to intangible cultural heritage martial arts in universities, curriculum construction can only provide students with brief insights. The inheritors of intangible cultural heritage martial arts believe that they cannot influence other martial arts courses (such as Tai Chi, competitive martial arts, Changquan, etc) in universities during their appointment. In addition, some universities have introduced numerous intangible cultural heritage martial arts projects, resulting in most inheritors having only two classes scheduled, all taking the form of theoretical courses. Only a few boxing styles have more teaching time scheduled because their cultural system is relatively complete, and their inheritors also have a high reputation in the martial arts industry or the local area. Inheritors of intangible cultural heritage martial arts express concerns about class hours, because the universities offer other martial arts courses in addition to intangible cultural heritage martial arts, and these courses should be the mainstream curriculum. So, the entry of intangible cultural heritage martial arts should not affect the mainstream curriculum. This shows that the construction of course content depends on the mutual recognition between universities and inheritors. On one hand, some intangible cultural heritage martial arts have a complete boxing system and high educational value; therefore, inheritors are unwilling to bring this system to universities for instruction. On the other hand, universities do not have a deep understanding of intangible cultural heritage martial arts and cannot recognize their cultural significance. They will not introduce intangible cultural heritage martial arts into universities, and schools focus more on the cultural style of martial arts. In the construction of course content, universities view the introduction of intangible cultural heritage martial arts into universities as mainly cultural inheritance, representing traditional Chinese culture. Most importantly, some inheritors of intangible cultural heritage martial arts believe that the characteristics of boxing should be reflected:

“*The school turns us into professors and visiting professors, and then allows each sect to conduct so-called field investigations. The aim was to tell the students: Do you know which martial arts schools and characteristics there are in here? So each inheritors was to talk about the characteristics, styles, origins, and so on. It's impossible to talk about things like Tai Chi, Xingyi Quan, because it's a big school. If you talk about it again, you'll just draw a snake and add more feet. You don't have any unique features*” *(On 10 January 2023, Field Diary, CCR-F08).*

Higher education institutions attract a large number of students; they are also centers for cultural exchange between different regions in China and even internationally. However, for students, this knowledge is unsurprising and uninteresting. Therefore, the requirement for intangible cultural heritage martial arts is a unique cultural style to supplement universal culture from the perspective of cultural specificity. As the inheritors of intangible cultural heritage martial arts have said, “Tai Chi and other major schools are like adding to the snake's feet without any distinctive features.” This also reflects the universities’ cultural preferences in constructing the content of intangible cultural heritage martial arts content. In addition, content selection based on base construction not only focuses on the cultural characteristics of intangible cultural heritage martial arts in curriculum construction, but also meets the needs of base construction.

“*Let me tell you the higher school how to operate. Firstly, it hires more experts in intangible cultural heritage as guest professors and categorizes them to explain cultural aspects. When the students can't even understand horse stance, bow stance, and rest in public physical education classes, the university calls the city level intangible cultural heritage inheritors to teach. If the classes are important, the school will let me, a master level person, give it public physical education classes. There are many students, and hundreds of students will take part together. The school will take photos and take photos. The main purpose is to provide materials to meet the scrutiny of several brands. Without these, the school cannot achieve its current scale in martial arts*” *(On 9 January 2023, Field Diary, CCR-F06).*

“Classifying teaching” is a fundamental principle in content construction to meet the needs of students at different levels. However, due to the fact that intangible cultural heritage martial arts are mainly based on regional martial arts, students in both professional and public sports classes are not familiar with intangible cultural heritage martial arts. Compared to students in public sports classes, students in professional classes have a certain foundation in martial arts and are more likely to complete their movements. Therefore, universities will use intangible cultural heritage martial arts as a supplement to professional classes. For students in public physical education classes, due to the large number of students and the large settings, only master level individuals can meet the needs of this inheritance. Because master level individuals are knowledgeable and experienced, they will make timely adjustments based on the specific situation of the students. More importantly, master level individuals can simplify the complex content of intangible cultural heritage martial arts and allow students who have never practiced or been exposed to martial arts in these public sports classes to better understand the content of intangible cultural heritage martial arts. For example, complex traditional martial arts concepts, such as meaning, Qi, and martial arts can be explained to students in just a few words by master level inheritors, while ordinary inheritors of intangible cultural heritage martial arts lack the ability to summarize and simplify these concepts. From this perspective, the construction of intangible cultural heritage martial arts inheritance content primarily based on base construction is a comprehensive consideration of the inheritor's ability, teaching objectives, and school requirements. This also reflects the complexity of curriculum construction in the intangible cultural heritage martial arts universities. It can be said that such universities are more complex in content construction because they do not fully accept the inheritance content of intangible cultural heritage martial arts, but rather adopt a “critical absorption” and “resource integration” approach. An in-service martial arts teacher from a martial arts university evaluates the inheritance content of intangible cultural heritage martial arts as follows:

“*We will turn intangible cultural heritage projects into a book as a tutorial. Just to say, the first step was to refine these intangible cultural heritage movements. The second step was centered around XX Fist, and then combined with other martial arts to add it up. If you were to focus solely on each intangible cultural heritage fist type, it would be too much. There are seven or eight schools, and class hours are not allowed. In this way, we have refined them for the sake of inheritance and promotion. Each of their fists has its own characteristics, as well as its own essence, and there are many repetitive movements. Both in terms of content and layout, they are not very clear, so they are passed down through this refinement method. Inviting the inheritors to come is definitely very grateful to them, but we cannot simply promote one. The inheritance must take its essence, discard its dross, and carry out inheritance. Only in this way can there be development and innovation. Only through innovation, improvement, and development can your boxing be inherited and promoted. The integration of intangible cultural heritage martial arts has been recognized by the inheritors of intangible cultural heritage martial arts. We will always cooperate with the inheritors of intangible cultural heritage, not that we will ignore them. No*” *(On 11 January 2023, Field Diary, GXJS-01).*

School education is a public temporal and spatial field for carrying out educational and inheritance activities; however, the spatial and temporal attributes of the public activity field will not change. In a constant temporal and spatial field, coordinating diverse educational resources is important. On one hand, universities with these rich educational resources should consider a balance between martial arts from on and off campus, rather than favor a particular genre. Therefore, universities adopt the approach of “extracting the essence” to highlight the “innovative” inheritance of intangible cultural heritage martial arts by deleting the repetitive actions in some boxing styles and extracting the essence from various schools. However, this also highlights the powerful role of curriculum content construction in inheritance of intangible cultural heritage martial arts universities. At the same time, it also shows that universities regard competitions as a criterion for evaluating the quality of intangible cultural heritage by placing intangible cultural heritage martial arts in the current field of competition and testing their efficacy. This shows that the inheritance content of intangible cultural heritage martial arts has been re-refined by university martial arts teachers to form their own curriculum systems. The above is only a preliminary screening in the construction stage of inheritance content. After teaching, university teachers will also conduct in-depth analyses of the inheritance content of intangible cultural heritage martial arts in universities for students. The purpose is to help students re-understand the inheritance content of intangible cultural heritage martial arts according to the competition rules:

“*The inheritors of intangible cultural heritage martial arts teach their own. But later on, we will explain to the students how to dissect this movement. You need to give them guidance, and tell the students how to appreciate and appreciate it. The routine did not change technically, but I changed my requirements. It means that the inheritors of intangible cultural heritage martial arts are not God. We will tell students not to change their style and content, but to make demands, such as the requirements for strength and rhythm, and how to reflect them in terms of spirit. If the inheritors of intangible cultural heritage martial arts talk about the meaning of attack and defense, I will explain why they did this action, where the meaning of attack and defense is, and how to express it. That is to say, after leaving you, I want to promote it and must require students to do it according to the current requirements. In competition, If I wanted to achieve good results, I had to practice according to the rules. I went to the competition a few times and followed the rules and requirements to do it. As an athlete, I have to adapt to this rule*” *(On 11 January 2023, Field Diary, GXJS-01).*

This demonstrates that the inheritors teach students “authentic” intangible cultural heritage martial arts; however, university martial arts teachers also need to conduct a “secondary analysis” according to the rules of martial arts competitions, and “re-examine” the inheritance content of intangible cultural heritage martial arts inheritors to ensure it meets the requirements of these competitions. In this context, martial arts teachers in university are like “football goalkeepers” or “a filter.” They re-screened the intangible cultural heritage martial arts according to specific competition rules.

Although the original ecology of the inheritance content in intangible cultural heritage martial arts universities may undergo certain changes under specific conditions, the inheritors of intangible cultural heritage martial arts adhere to the preservation of the unique style of boxing. The inheritor of Songxi Fist, a provincial-level intangible cultural heritage project, regards maintaining the style of the martial arts as the original intention of university inheritance, and believes that the construction and explanation of inheritance content must be vivid. “If you want to go to school for inheritance, regardless of any martial arts, you need to retain your unique style of the martial arts. Firstly, it must have a coherent history, and secondly, it must have unique characteristics of the sect. You need to embody the essence of tradition. When entering school, you must inherit tradition, which is a prerequisite and the overall direction. The first is the truth, and the second is the key. Students should be willing to listen, and all your theories need to be combined with practice” (11 January 2023, Field Diary, CCR-05).

A distinct boxing style is the soul of any boxing sect. Particularly for intangible cultural heritage martial arts, highlighting the style of boxing is key to the development of boxing. Without a unique style, any boxing form will eventually be assimilated by other boxing styles. Regarding the inheritance of martial arts in universities, the lack of style in martial arts means a lack of cultural characteristics. It also means that the general sense of culture cannot meet the role of cultural supplementation, so there is no need for universities to introduce it. Because the general culture is familiar to everyone and lacks its own characteristics. That is to say, the boxing style represents the cultural charm of intangible cultural heritage martial arts, which is not just a superficial disguise or forgery, but has a cultural foundation emanating from the bones of boxing. From a cultural perspective, any intangible cultural heritage martial arts project has core skills that have been passed down through generations. These core memories can span time and space, forming the style of the martial arts genre, and are iconic skills that distinguish between martial arts genres. Any inheritor of intangible cultural heritage martial arts is a spokesperson for the style and characteristics of the martial arts genre. Therefore, regardless the changes in transmission methods, they always adhere to the style of the martial arts genre and uphold its “traditional coherence and unique style.”

Although maintaining the style of intangible cultural heritage martial arts boxing is a basic concept for inheritors in university settings, it does not mean that the more profound the boxing, the better. The depth of boxing style or theory does not necessarily represent the quality of intangible cultural heritage martial arts university inheritance. On the contrary, the strategy adopted by inheritors of intangible cultural heritage martial arts in the construction of inheritance content is to “avoid the heavy and neglect the light,” eliminate the profound and difficult to understand content, and teach some universally applicable boxing principles, so that students can “learn” and “understand” them at a glance. Essentially, whether it is intangible cultural heritage martial arts or another combat technique, it is a technical means of using the body to produce significant impact through the reasonable coordination of the hands, elbows, shoulders, feet, legs, hips, and other bodies. Inheritors compare and create connections of this technique in the selection of inheritance content to enable students to have a simpler understanding of the principles of intangible cultural heritage martial arts. However, this does not mean that intangible cultural heritage martial arts have lost their characteristics, but rather indicates that the inheritors have a broad perspective and the ability to distinguish differences from similarities. Therefore, as stated by the inheritors of intangible cultural heritage martial arts, “not only should the movements be standardized, but also able to be used”; “practice for use” reflects the practical thinking in the inheritance of intangible cultural heritage martial arts in universities. This is also a key factor that inheritors of intangible cultural heritage martial arts should consider when selecting inheritance content. The inheritor of intangible cultural heritage martial arts narrated the teaching scenes of martial arts in universities, which can be said to be “unique,” and gave students a live demonstration of “whether traditional martial arts can be fought”:

“*I talked for an hour, about ‘last come, first served.’ In layman's language, it is called defensive countermeasures. I said you are all professional classes, including practicing Taekwondo, martial arts, and Sanda. ‘Come out a few people and have a fight with me.*’ *Four people were called up. A sanda student who is 1.8 m tall. I said whatever you want to do, I won't shout for your left foot. As soon as he stepped out, I charged in and took a step in. I shrugged and exerted force, knocking him down. Sprinkle and fall to the ground, fly into the crowd and sleep (lie down with four feet facing the sky). Then, I said* ‘*come and go,’ then another three students won't come and immediately return to their positions and sit up*” *(11 January 2023, Field Diary, CCR-F05).*

Intangible cultural heritage martial arts are a form of expression for body techniques, acting as symbols representing culture. Therefore, it is only through this immersive on-site teaching that students can truly appreciate the charm of this physical technique. One reason why the inheritors of intangible cultural heritage martial arts emphasize the combat effectiveness of these techniques is partly because the boxing technique itself has strong combat effectiveness. Although the late start system reflects the common combat methods in traditional martial arts, the practical teaching of specific boxing techniques can better enable students to experience the charm of boxing. On the other hand, due to the personalities of the inheritors, this teaching method better reflects their characteristics. Another aspect is that the traditional martial arts have been heavily influenced by negative connotations, and the inheritors have used this to prove the strong style of traditional martial arts. These factors collectively contribute to the practicality of how inheritors of intangible cultural heritage martial arts select and explain inheritance content. This way, students can at least experience the combat attributes of traditional martial arts.

## 5 Discussion

Based on the above results, it can be concluded that the following characteristics in the inheritance of intangible cultural heritage martial arts in universities are worth further discussion.

### 5.1 Highlighting local knowledge of cultural heritage

Local knowledge, a concept pioneered by contemporary American anthropologist Clifford Giltz, has had a profound impact on various fields, including philosophy and linguistics (43). Local knowledge and universal knowledge are corresponding concepts; the former highlights the local characteristics of knowledge system construction and has a strong substantive color, while the latter emphasizes the universality of knowledge system construction, representing formal thinking. In this sense, local knowledge emerged from the “methodological struggle between universalism and historical particularism,” “global modernization,” and “postmodern trends,” forming the concepts of “anthropology represented by Giltz” and “philosophy of scientific practice” (44). Locality, relativity, and particularity are prominent features of local knowledge: “it is a knowledge system that is generated in a certain context (such as historical, regional, ethnic, racial, etc.) and is confirmed, understood, and protected in that context” (45); “it is not a set of established and proven propositions, but a set of activities or practical processes” (46). In other words, local knowledge is the cognitive ability formed in specific object-oriented practices, and this cognitive ability has certain situational and special characteristics.

During the process of constructing curriculum content, the inheritance of intangible cultural heritage martial arts in universities places more emphasis on its local knowledge characteristics. It is not only the externalization of its own attributes in university inheritance, but also the reconstruction of its own attributes in university inheritance. On the one hand, intangible cultural heritage martial arts have already formed a local knowledge system in their own construction before being practiced in universities. This system is based on the natural resources, material environment, and humanistic spirit of regional culture. It is an internal regulation that maintains its own cultural style to a certain degree of cultural constancy and persistence and is also a source of life that distinguishes itself from others. In this system, local cultural knowledge plays a role in the cultural construction inherited by universities, forming a curriculum system with local characteristics. As Professor Chen Zhenyong said, “Cultural reconstruction is the contemporary value reproduction of regional martial arts culture research” (47). As a form of local knowledge with regional cultural attributes, intangible cultural heritage martial arts play a role in reproducing contemporary values in the curriculum construction of university inheritance; this function is also essential to regulating cultural inheritance. The cultural inheritance of intangible cultural heritage martial arts in universities aims to transmit the accumulated cultural power to students, thus achieving intergenerational cultural continuity through the educational mechanism. On the other hand, the inheritance of intangible cultural heritage martial arts in universities is influenced by the new teaching environment. It is necessary to redefine curriculum construction and achieve innovative transformation in the inheritance of intangible cultural heritage martial arts in universities. However, this innovative transformation is based on its own characteristics.

Regarding the integration of intangible cultural heritage martial arts in universities, although it has entered universities from the private sector and the educational environment has changed, the introduction of intangible cultural heritage martial arts into universities is a cultural identity that supplements the existing school martial arts education. Whether it is the purpose, object, method, or content of inheritance that differs from traditional martial arts education, it is a culturally centered martial arts inheritance. Although intangible cultural heritage martial arts has not changed, the focus of university inheritance has shifted from physical fitness to intellectual and emotional development. In other words, “culture” is the ultimate goal of intangible cultural heritage martial arts in universities. This is also why intangible cultural heritage martial arts highlight its local knowledge in the inheritance of universities. Local knowledge represents the deepest and most simple aspects of traditional culture. In this sense, curriculum construction in the inheritance of intangible cultural heritage martial arts in universities is also a reconstruction of local knowledge. Local knowledge is a concept of inheritance, and the specific implementation of this cultural concept requires intangible cultural heritage martial arts to highlight its core technical skills in university inheritance.

### 5.2 Highlighting the core craftsmanship of body techniques

Body technology is a concept suggested by French sociologist Moss, who believes that “the body is the first and most natural tool for humans, or rather the first and most natural technological object and means” (48). In this sense, the body serves as a technology that reflects the cultural concept of the body as a tool or instrument. From the Chinese perspective of the body, it is considered the foundation of human existence. As Zhang Zailin said, “If Western philosophy is based on consciousness, then ancient Chinese philosophy is based on the body” (49). Essentially, intangible cultural heritage martial arts is one of physical movement, but the body plays an important role. At the same time, physical exercise is influenced by cultural beliefs. The physical attributes of these martial arts determine the instability and heterogeneity of the techniques of different practitioners. On the one hand, this characteristic offers infinite possibilities for the reconstruction of intangible cultural heritage martial arts body techniques; on the other hand, it creates obstacles in maintaining the cultural style of boxing.

In universities, the aim of inheritance of intangible cultural heritage martial arts is to use body technology to convey traditional Chinese culture. However, due to the variability and individuality of body techniques, the inheritors must emphasizes more on the core craftsmanship of its body techniques in the process of curriculum construction. Their main aim is to maintain the style of boxing and the characteristics of traditional culture, and limit the influence of body deformation. First, there is integrated creation through reorganizing the core skills of dozens of boxing equipment routines in this boxing genre, selecting representative technical movements that can represent the style of this boxing genre, and creating new representative routines to ensure the technical characteristics of the boxing genre. Second, there is transplantation for reference. This form of curriculum construction mainly occurs in universities that lack inheritors of intangible cultural heritage martial arts and where ordinary university teachers assume the role of inheritors. For them, they are not the inheritors of intangible cultural heritage martial arts, so they need't undergo the stage of innovative transformation. Instead, they directly use a nationally recognized intangible cultural heritage martial arts as the curriculum content. Third, there is direct transplantation, which is the curriculum construction of intangible cultural heritage martial arts in universities that does not integrate and create routines for college students. The mature and established routines in the school are used as course content, and selection is based mainly on students’ interests, needs, majors, and employment prospects. However, regardless of the way intangible cultural heritage martial arts are constructed in universities, they all have the same goal: to highlight the core skills of intangible cultural heritage martial arts; to distinguish them from the established martial arts content in university education; to highlight the value of their traditional culture; and to emphasize their cultural heritage.

### 5.3 Emphasizing the cultural tradition of physical skills

The cultural tradition is closely related to Chinese culture, Chinese traditional culture, and excellent Chinese traditional culture, and there is a certain inherent relationship between them. In Li Zonggui's view, Chinese culture and traditional Chinese culture are different expressions of the same concept. Chinese culture is formed based on the national significance of Chinese traditional culture, and the two are consistent in connotation. Excellent traditional Chinese culture, a subset of Chinese culture, reflects the healthy spiritual direction of Chinese culture, inspires people to move forward, inspires national self-confidence and pride, has the function of national cultural identity, historical inheritance and stability, and still has strong vitality today (50). In this sense, the traditionality of culture is a mainline that connects Chinese culture to inherit both the past and the present in historical changes, and then connects the future. For those who are immersed in culture, their understanding of cultural tradition belongs to the subconscious level of cultural identity, and they only become aware when the cultural environment undergoes changes. As Liu Kuili said, the recognition of cultural traditions, just like water to fish and air to birds, is natural and indifferent. Only when a nation faces the impact of different cultures, or when a person is immersed in another cultural system, will they explore and experience the value of cultural traditions and generate a strong sense of identity (51). It can be inferred that the presentation of cultural tradition requires certain historical conditions and the cultural consciousness of the parties involved.

For the inheritance of intangible cultural heritage martial arts in universities, cultural tradition is a valuable cultural quality. In the course of historical changes, due to its consistent cultural style of traditional martial arts, it has become more imbued with the style of excellent traditional Chinese culture, which is also the value and significance of its entry into universities for inheritance. In other words, the inheritance of intangible cultural heritage martial arts in universities is based on highlighting the traditional nature of culture. Without it, the value of its inheritance would be lost. From a physical education perspective, there are numerous and exquisite teaching projects in physical education in schools, and students have many opportunities to choose projects, including various compulsory, elective, and limited elective courses, as well as other martial arts projects. However, a common goal of these projects is to promote the physical and mental health of students. There are few projects that truly inherit culture, which is determined by the cultural attributes of the projects themselves in physical education in schools, differing from intangible cultural heritage martial arts. Its main purpose is not only to promote the physical and mental health of students, but more importantly, to teach traditional Chinese cultural values. Cultural inheritance is the main theme of its introduction into universities. This is also an important reason for inheritors of intangible cultural heritage martial arts to adhere to cultural tradition in curriculum construction. More importantly, the main aim is to construct a system curriculum cultural symbols. From this, it can be seen that highlighting the cultural tradition of intangible cultural heritage martial arts in university is the foundation, and it is also the fundamental basis for the long-term development of intangible cultural heritage martial arts in university. As Wang Gang and Qiu Pixiang stated, “Innovation without tradition is pale and without foundation; only by paying attention to tradition, learning from tradition, and applying tradition can tradition continue to flow” (52).

## 6 Conclusion

The aim of this study was to explore the process of integrating intangible cultural heritage martial arts into the modern school education system from an educational ethnography perspective. According to the nature of curriculum construction, the curriculum construction for the inheritance of intangible cultural heritage martial arts in universities can be divided into three categories. The first category is the curriculum construction mainly composed of teachers on campus. The inheritors of this type of curriculum construction are relatively complex and can be divided into inheritors with a teacher-teacher relationship with the public and in-service teachers in universities (including martial arts teachers and other physical education teachers). The former, due to their relationship with the public, has a relatively complete skill system for intangible cultural heritage martial arts; therefore, the curriculum of inheriting intangible cultural heritage martial arts in universities has been reorganized to form a curriculum with characteristics of university inheritance. The latter do not have a teacher-student relationship with inheritors of intangible cultural heritage martial arts in folk. The curriculum construction of inheriting intangible cultural heritage martial arts in universities adopts the method of directly transplanting intangible cultural heritage martial arts content into the normal curriculum system. Therefore, the curriculum construction belongs to direct transplantation rather than creative utilization. The second category is curriculum construction dominated by off-campus inheritors who mainly come from the public arena. They tend to focus more on local knowledge in curriculum construction and construct a curriculum system that belongs to the local curriculum. Their main principle when selecting course content is to meet the professional needs of students and inherit excellent traditional Chinese culture. Curriculum construction is not about rebuilding the boxing system and integrating the inheritance content, but about selecting or intercepting boxing routines that are suitable for the characteristics of college students from existing boxing styles to enrich the course content. The third category of curriculum construction is centered around base construction, and the inheritors of this type of curriculum construction are the most complex among the three types of curriculum construction. The inheritors of curriculum construction include both external inheritors and internal inheritors, as well as in-service teachers in universities. Therefore, curriculum construction is the product of the three categories interacting. However, regardless of the type of curriculum construction in the inheritance of intangible cultural heritage martial arts in universities, the curriculum content exhibits local, core, and traditional characteristics to form a university curriculum system that is different from the folk inheritance system. These curricula can effectively compensate for the cultural deficiencies in modern school martial arts education and also play a role in continuing China's excellent traditional culture. Among them, locality is determined by the regional characteristics of intangible cultural heritage martial arts, and the core emphasizes the core skills of intangible cultural heritage martial arts, which are determined by the style of boxing. Tradition is the cultural value of intangible cultural heritage martial arts, which is determined by the historical genes of intangible cultural heritage martial arts.

## 7 Practical applications

Based on the above conclusions, this study further discusses how traditional Chinese martial arts can be integrated into the school education system. Chinese martial arts is a cultural symbol system formed on the basis of traditional Chinese culture, playing an important educational role in the historical process. Since modern times, Chinese martial arts have gradually embarked on a journey of modern school martial arts education under the influence of modern Western sports ideas. However, in this process, how to integrate traditional martial arts culture into the modern educational system has become an important research question, and a unified answer has not yet been formed. A study has pointed out that “martial arts education entering schools should be mandatory and not allowed to be chosen by students, because students lack recognition of the competition of national culture” (53). Although incorporating martial arts into the modern educational system through mandatory means can have a certain effect, this approach can only solve temporary problems and cannot break through the inherent vitality of martial arts education, which ignores the subjectivity of students. From the essence of education, the vitality of school martial arts education comes from the inherent quality of martial arts, which fundamentally implements the educational goal of putting people first. Through compulsory means, the student-centered teaching purpose is ignored, which is not conducive to the integration of traditional martial arts culture into the modern curriculum system. Since the establishment of the People's Republic of China, Chinese martial arts has always been an important part of the school physical education curriculum, but it has never truly solved the difficulties in its education. It can be seen that simply using compulsory means cannot fundamentally solve the problem of integrating traditional martial arts culture into modern educational systems. When exploring the implementation path of protecting and inheriting traditional martial arts, Wang Gang and others believe that the folk inheritance methods of traditional martial arts should be combined with modern educational inheritance, the educational awareness of traditional martial arts should be strengthened, the originality in the inheritance process of boxing should be highlighted, and the cultivation of traditional martial arts elites should be placed on higher education, breaking through the inherent educational model (54). Another study also suggested that choosing boxing as content of the school martial arts education is a top strategy, because it has touching stories, a minimalist technical start, profound theories, and a systematic culture that runs through it (27). These studies emphasize the educational awareness, originality, technical simplification, profound theory, and important role of higher education in cultivating traditional martial arts talents but have not yet been proven in practice. This study provides further practical evidence for the integration of traditional martial arts into the school education system, and also verifies the feasibility and effectiveness of adhering to the traditional characteristics of boxing.

When discussing the ways in which traditional martial arts can be integrated into the modern school education system, Liu Wenwu puts the key to the construction of martial arts teachers. He advocates that the content of talent cultivation in sports colleges should shift from competitive martial arts to traditional martial arts, and that university martial arts teachers should delve into folk customs and improve teaching management departments (34). Some scholars also believe from the perspective of communication studies that the educational function of martial arts is different from other sports, as well as from Chinese language, history, and moral education. It is an organic unity of cultural education and physical education (55). Starting from the characteristics of traditional martial arts themselves, these views have profound insights. The feasibility of traditional martial arts is determined by the martial arts teachers, and the unique advantages of traditional martial arts in school education can also be leveraged. However, these views have not yet taken into account the spatial environment and field characteristics in which school martial arts education plays a role. In other words, the inheritance of intangible cultural heritage martial arts in universities should not transfer traditional martial arts skills and culture to universities but should go through technological transformation and innovation. The results of this study show that the inheritance of intangible cultural heritage martial arts in universities is a simplified, core, and traditional cultural inheritance. It is based on the skill reconstruction in the educational context of universities to embody the characteristics and cultural style of traditional martial arts rather than a simple transplantation of traditional martial arts from the public to universities. In this sense, the core of how traditional martial arts are integrated into the modern school education system should not only focus on the role of inheritors of intangible cultural heritage martial arts, but also on cultivating innovative talents with condensed characteristics of intangible cultural heritage martial arts and high-level “coach type” talents who effectively convey the characteristics of intangible cultural heritage martial arts to relevant teachers. This is the current and future key to integrating traditional culture into the modern school education system. It is also a guarantee for establishing the continuity of Chinese national culture through school mechanisms.

## Data Availability

The original contributions presented in the study are included in the article/Supplementary Material, further inquiries can be directed to the corresponding author.
